# Alteration in body water compartments following intermittent fasting in Ramadan

**DOI:** 10.3389/fnut.2023.1232979

**Published:** 2023-08-14

**Authors:** Mohammad Taghi Najafi, Ali Sadoogh Abbasian, Hamed Mohammadi, Mohammad Reza Abbasi, Mohammad Reza Khatami, Ali Ghafari, Mohammad Hossein Shojamoradi

**Affiliations:** ^1^Nephrology Research Center, Tehran University of Medical Sciences, Tehran, Iran; ^2^Department of Internal Medicine, Arak University of Medical Sciences, Arak, Iran; ^3^Department of Clinical Nutrition, School of Nutritional Sciences and Dietetics, Tehran University of Medical Sciences, Tehran, Iran

**Keywords:** body composition, Ramadan, intermittent fasting, body water, weight loss

## Abstract

Concerning the health outcomes of intermittent fasting in Ramadan, loss of fat-free mass (FFM) and changes in the content of body water are of paramount importance. In this study, we aimed to assess the concomitant alterations in body water compartment and composition following Ramadan fasting in healthy individuals. We conducted an open-label cohort with longitudinal follow-up, involving 73 healthy medical staff who planned to fast for at least 20 consecutive days during Ramadan. The primary outcomes of the cohort were changes in parameters related to body composition and water content, which were measured using bioelectrical impedance analysis by InBody S10 (InBody, Seoul, South Korea). Based on the results, the participants’ weight decreased significantly by approximately 1,030 g after the fasting period (*p* < 0.001). There was a significant reduction in the fat mass of an average 828 g (*p* < 0.001), which accounted for more than 80% of the weight loss. The decline in FFM was not significant (190 g; *p* = 0.234). The amount of total body water (TBW) and extracellular water (ECW) did not change, while intracellular water (ICW) decreased significantly by about 160 mL (*p* = 0.027). A strong correlation was observed between the reduction of phase angle and the increase in ECW/TBW ratio (*R* = −0.71, *p* < 0.001). Overall, our findings revealed a minimal amount of weight loss after Ramadan fasting, which was mainly due to the loss of fat mass. The parallel decrease in ICW and phase angle indicated impaired cell membrane integrity, with subsequent movement of water from the intracellular to the extracellular compartment.

## Introduction

Intermittent fasting is described as an interventional strategy for weight loss, with alternating periods of fasting and feasting (1). This type of fasting can restore cellular homeostasis, increase antioxidant defense, and suppress inflammation; these alterations ultimately improve cardiovascular health and exert beneficial metabolic effects (2–4).

Although in recent years, the popularity of dietary regimens based on intermittent fasting has grown, intermittent fasting during the month of Ramadan has been practiced by many Muslims for many years. Each year, in the ninth month of the lunar calendar, Muslims abstain from food and fluid intake during daylight hours for 29–30 consecutive days. The fasting window usually begins with a meal before sunrise (“Suhoor”) and ends with a meal after sunset (“Iftar”). The duration of fasting varies from about 12 to more than 18 h, based on solar season and geographic location (5).

The effects of Ramadan fasting on various aspects of health, including metabolic parameters, blood pressure control, sleep quality, and physical performance, have been well investigated in previous researches (6–9). Most of these effects are attributed to the reduced calorie intake of individuals and the consequent alterations in body weight and composition due to fasting (10). Nonetheless, studies evaluating the alterations in body composition due to Ramadan fasting have yielded inconclusive results. While the majority of these studies have reported a significant decrease in weight and fat mass following Ramadan fasting, others have failed to show any significant changes (11). Such heterogeneity may be attributed to several factors, such as the amount of calorie intake in the feasting window, duration of fasting, and cultural rituals affecting the intake of macro-and micronutrients. In this regard, a meta-analysis by Jahrami et al. showed that intermittent fasting in Ramadan conferred a significant, albeit small, amount of weight loss, which was associated with the duration of fasting (12).

Although some minor fluctuations may occur in the amount of body water, it remains relatively constant during a normal life. Abstinence from water and other beverages during the fasting window in Ramadan may result in some degree of hypovolemia and cause subsequent symptoms, such as headache, impaired cognition, and decreased physical performance (13–15). Besides, the amount of fluid intake in the feasting window may be below the recommended level (16). Very few studies have directly investigated alterations in the content of body water following Ramadan fasting. In a study using deuterium oxide as a water tracer, Leiper et al. revealed that the total body water (TBW) content was conserved during Ramadan (17). Additionally, in a bioimpedance analysis, Alinezhad-Namaghi et al. documented a significant, but small amount of loss in the TBW content (<1%) after Ramadan fasting (18). Other studies have investigated indirect measures, such as urine and plasma osmolarity, urine flow rate, and hematocrit and serum creatinine levels to assess alterations in the body water content during Ramadan fasting (15, 19–21).

Concerning the health outcomes of Ramadan fasting, two main alterations need to be addressed; one is the reduction of fat-free mass (FFM), which reflects the depletion of body cell mass, and the other is the alteration of body water compartments, leading to hypovolemia and related complications. While changes in the body composition following Ramadan fasting have been extensively reported, alteration in the water content of body has not been well documented in previous studies. Furthermore, the relationship between the changes in body composition and water content is not yet clear. Thus, in this study, using the validated method of bioimpedance analysis, we aimed to assess the alterations in body water compartments and composition following Ramadan fasting in the healthy medical staff of our hospital.

## Methods

### Ethical considerations

The study protocol was designed according to the Declaration of Helsinki and approved by the local ethics committee of our institute (IR.TUMS.IKHC.REC.1399.324). All participants signed written informed consent forms before entering the study.

### Study design and population

The study followed an open label longitudinal follow-up design with convenient sampling to compare anthropometric indices, body composition and water content at two points: baseline and after Ramadan fasting. The study population consisted of the healthy medical staff of Imam Khomeini Hospital Complex (Tehran, Iran). Participants were recruited via an e-mail from the secretary’s office of the hospital. Adult individuals who planned to fast for at least 20 consecutive days in Ramadan were included if they provided written informed consent forms for participation in the study. The exclusion criteria were: being affected by any acute or chronic medical conditions, having a cardiac electrical device implanted, and recent use of any nutritional supplement.

The study was conducted during May–June 2018 (Ramadan 1439 AH) in Tehran, Iran. In this year, Ramadan started on May 17 and finished on June 14. The average length of fasting was more than 16 h each day. The mean ambient temperature was about 23°C and the maximum daytime temperature rose from 25°C at the start of Ramadan to 35°C by the end.

### Sample size calculation

The sample size was calculated based on the primary outcome of weight loss. Assuming *α* = 0.05 and *β* = 0.8, about 66 participants were required to detect a weight loss of 700 g (22). At the beginning of the study, a total of 86 individuals were enrolled. Of this sample, 73 individuals were included in the study, while 13 were excluded due to failure to fast during Ramadan (*n* = 6) or failure to return for the second evaluation (*n* = 7).

### Anthropometric measurements

The anthropometric indices were measured for each participant during the week before Ramadan and then after 20 consecutive days of fasting during Ramadan. Body weight was measured using a calibrated electronic scale (Rasa, Tehran, Iran), wherein the subject wore light clothes with no shoes on, and measurements were recorded to the nearest 100 g. Moreover, height measurements were performed by a portable stadiometer (OTM, Tehran, Iran) while standing barefoot and rounded to the nearest 10 mm. To increase accuracy, the measurements were taken in the NPO state (“nothing by mouth”) before Ramadan and after midday during Ramadan, at least 8 h after the last meal.

### Measurement of body composition

The body composition was evaluated based on a bioelectrical impedance analysis by InBody S10 body water analyzer (InBody, Seoul, South Korea). This method was previously validated for the assessment of body composition in Iranian people (23). Generally, this device applies a small alternating current to the body via tetrapolar eight-point tactile electrodes and separately measures the impedance of the trunk, arms, and legs at six different frequencies, including 1, 5, 50, 250, 500, and 1000 kHz. Impedance at high frequencies represents the conductivity of the TBW compartment, while impedance at low frequencies relies on the conductive properties of the extracellular water (ECW) compartment. Additionally, FFM was estimated based on the TBW content, and thereafter, the fat mass was calculated (24). Moreover, the phase angle, defined as the ratio of electric reactance to electric resistance at 50 kHz, was calculated according to the phase angle formula (25).

### Statistical analysis

Statistical analyses were performed in SPSS Version 13 for Windows (SPSS Inc., IL, USA). Quantitative variables were reported by measuring the mean values and standard deviations, and qualitative variables were described by calculating frequency and percentage. A paired *t*-test was performed to compare numerical body composition variables before and during Ramadan fasting, if they were normally distributed. Moreover, Wilcoxon test was used for non-parametric analyses of data with a skewed distribution. To compare the mean values of variables between the two groups, an independent sample *t*-test or the Mann–Whitney *U* test was carried out if appropriate. Finally, relationships between continuous variables were evaluated using Pearson’s correlation coefficient test. A *p*-value less than 0.05 was considered statistically significant.

## Results

### Body weight changes

The mean age of the participants was 41.78 years, and 26% of them (*n* = 19) were male. The fasters were observed for an average of 22.4 days. Nearly 75% of the participants (*n* = 55) lost weight after Ramadan fasting, while about 15% (*n* = 11) experienced weight gain. On the other hand, the weight of nearly 10% of the participants (*n* = 7) remained unchanged.

The subjects’ weight decreased significantly by 1,030 g on average from the baseline (*p* < 0.001). The mean amount of weight loss was 1,380 and 0.890 g in male and female subjects, respectively, without any significant difference (*p* = 0.16). Similarly, no significant difference was observed between individuals older or younger than 40 years. Approximately two-thirds of the participants (*n* = 46) had an initial body mass index (BMI) of greater than 25 kg/m^2^. These individuals experienced a significantly higher mean weight loss than those with a BMI of less than 25 kg/m2 (1243 vs. 588 g; *p* = 0.046). Additionally, a significant correlation was observed between the initial BMI and the amount of weight loss (*R* = 0.239, *p* = 0.042).

### Body composition changes

Table 1 presents the weight and body composition of the study sample before and after Ramadan fasting, as well as the percentage of change in these variables. The participants significantly lost their fat mass following Ramadan fasting (828 g, 3.29%), while the amount of FFM reduction was not significant (190 g, 0.39%). According to the results, losses of skeletal muscle mass, protein mass, and body cell mass were all significant after the fasting period (Table 1).

**Table 1:** Alterations in the body composition, body water compartments, and 50-kHz phase angle after Ramadan fasting in the study.

	Before fasting (mean ± SD)	After fasting (mean ± SD)	Amount of change	Percentage of change	*p* value
Weight	74.53 ± 1.7	73.52 ± 1.7	1.019	1.29	<0.001
BMI	27.11 ± 0.4	26.73 ± 0.4	0.37	1.36	<0.001
Fat mass	26.63 ± 0.9	25.8 ± 0.9	0.828	3.29	<0.001
Percent of body fat	35.78 ± 0.9	35.11 ± 0.9	0.66	1.84	0.001
Fat free mass	47.9 ± 11.8	47.71 ± 11.65	0.190	0.39	0.234
Skeletal muscle mass	26.36 ± 0.8	26.15 ± 0.8	0.211	0.66	0.028
Total body water (TBW)	35.19 ± 1	35.07 ± 1	0.121	0.21	0.28
Extracellular water (ECW)	13.44 ± 3.13	13.48 ± 3.13	−0.04	0.29	0.39
Intracellular water (ICW)	21.75 ± 0.6	21.59 ± 0.6	0.161	0.61	0.027
ICW/TBW	61.71 ± 0.67	61.47 ± 0.64	0.24	0.39	<0.001
ECW/TBW	38.3 ± 0.65	38.53 ± 0.61	0.23	0.6	<0.001
Protein mass	9.39 ± 2.37	9.32 ± 2.34	0.078	0.83	0.018
Body cell mass	31.15 ± 7.88	30.92 ± 7.77	0.235	0.75	0.025
50-kHz driven phase angle	5.62 ± 0.07	5.49 ± 0.07	0.13	2.33	<0.001

### Changes in body water compartments

The mean values of TBW and ECW contents did not significantly change after fasting, whereas the intracellular water (ICW) content decreased significantly by almost 160 mL (Table 1). Moreover, the ICW/TBW ratio decreased from 61.71 to 61.47% after Ramadan fasting (*p* < 0.001).

### Changes in 50-kHz phase angle

**Figure 1:**
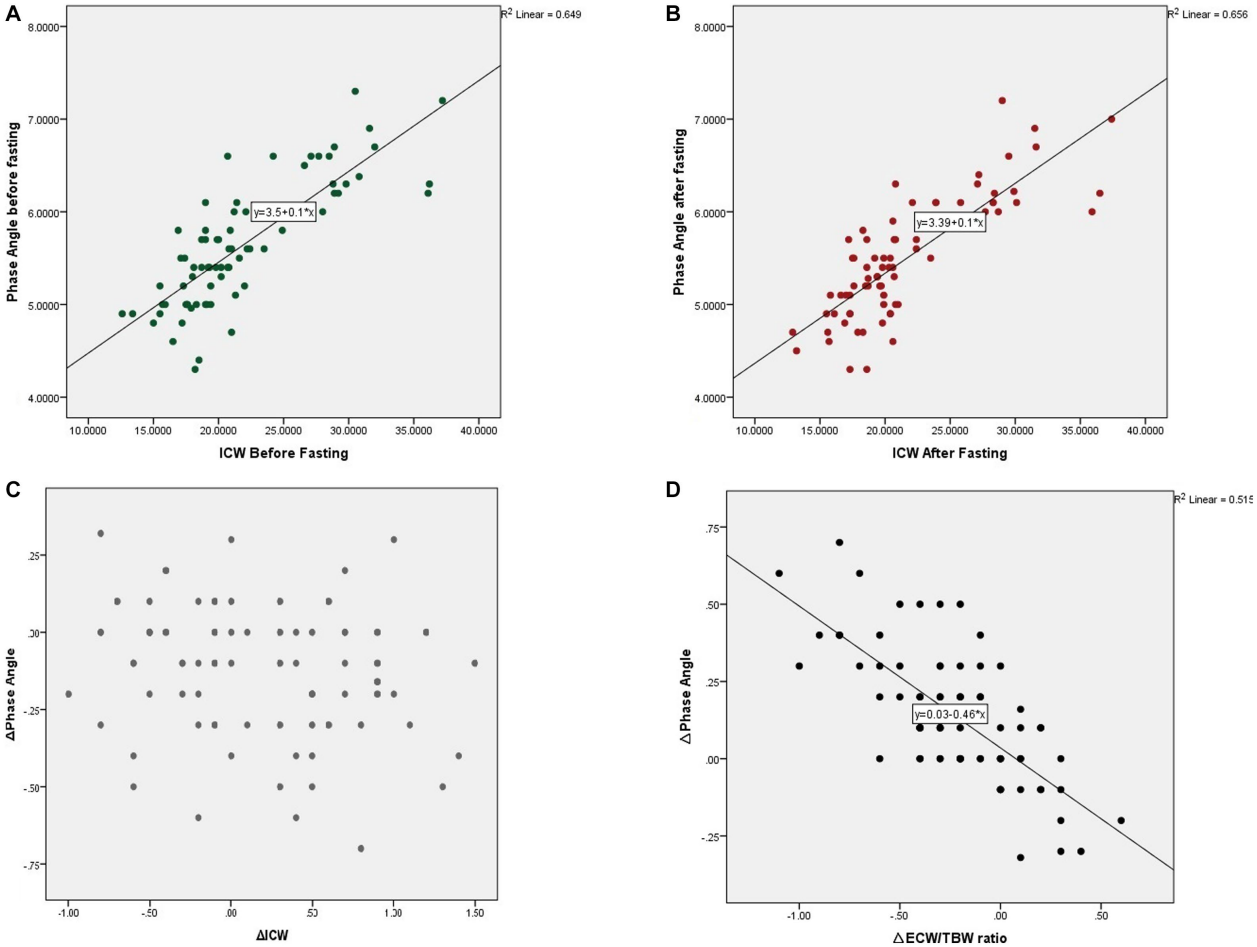
Correlation between 50-kHz phase angle and ICW before **(A)** and after Ramadan fasting **(B)**. The ∆phase angle and ∆ICW are not correlated **(C)**, but a strong correlation can be observed between the ∆phase angle and ∆ECW/TBW ratio **(D)** (ICW, Intracellular water; ∆phase angle, Alteration in 50-kHz phase angle after fasting; ∆ICW, Alteration in ICW after fasting; ∆ECW/TBW, Alteration in the ratio of extracellular body water to total body water after fasting).

The 50-kHz phase angle decreased significantly by about 0.13° (Table 1). The decrease in the phase angle was correlated with a reduction in the protein mass (*R* = 0.25, *p* = 0.03). Figure 1 shows the correlation between phase angle and ICW before and after Ramadan fasting, as well as the correlation between alterations in phase angle and ECW/TBW ratio following the fasting period. Although alterations in the phase angle and ICW were not correlated, a strong correlation was found between the amount of reduction in the phase angle and the increase in the ECW/TBW ratio (*R* = −0.71, *p* < 0.001).

## Discussion

Fasting in the month of Ramadan, characterized by diurnal time-restricted feeding, is an obligatory practice for healthy adult Muslims. This form of intermittent fasting is unique, since practitioners abstain from both food and fluid in the fasting window. It is estimated that annually, 1 billion Muslims follow intermittent fasting in Ramadan. Thus, Ramadan fasting can provide valuable insights into the effects of intermittent fasting on metabolic parameters and body composition. In this study, we sought to explore the concomitant effects of Ramadan fasting on body composition and water content.

### Highlights of principal findings

Following the participants for more than 22 days of fasting, we indicated a significant, albeit small, amount of weight loss. Utilizing a two-compartment model for the evaluation of body composition, our findings revealed that more than 80% of this weight loss was due to the reduction in fat mass. Conversely, the loss of FFM contributed to nearly 20% in body weight loss. These findings can explain the observed link between pre-fasting BMI and the amount of weight loss.

From a nephrologic point of view, ongoing water loss due to abstinence from fluid intake can result in some degree of volume depletion during Ramadan fasting. In this study, the month of Ramadan was in the late spring with a relatively high ambient temperature. Despite such environmental conditions, our findings highlighted two important points; unchanged levels of TBW and a significant decrease in ICW content. These alterations ultimately led to an increased ECW/TBW ratio after the fasting period.

The phase angle, defined as the ratio of reactance to resistance, represents body cell mass, cell membrane integrity, and cell function (25). Despite negligible decrease in FFM, we found a significant reduction (2.33%) in the 50-kHz whole body phase angle, which was in parallel with the amount of loss in protein mass. Therefore, phase angle can serve as a more sensitive marker for nutritional assessment in Ramadan fasting.

### Comparison with other studies

In terms of weight loss, our findings are consistent with the results of most previous studies, which showed a transient reduction in weight following Ramadan fasting. A meta-analysis by Jahrami et al., reported that diurnal intermittent fasting in Ramadan led to a weight loss of about 1,022 g (12). Consistent with our results, age and sex were not related to the amount of weight loss. Unlike the study by Norouzy et al., our population was heterogeneous in terms of weight loss, and even some cases experienced weight gain after the fasting period (26). Several factors may underlie this heterogeneity, including daily physical activities, amount of calorie intake in the non-fasting window, and different dietary habits that need to be evaluated in future studies. In line with our study, a meta-analysis by Fernando et al. indicated an association between the pre-Ramadan BMI and the amount of weight loss (27). Regarding weight loss, Ramadan fasting may be more effective in individuals with a higher pre-Ramadan BMI.

Although the proportional loss of fat mass to FFM may provide important clues about the safety of weight loss strategies, the amount of excessive loss in FFM and its safe level remain unclear. A study by Alinezhad-Naghami et al. showed that 76% of weight loss was attributed to the loss of fat mass (18). In another study by Syam et al., the reduced body fat had the greatest contribution to weight loss, while unlike our study, the protein mass remained unchanged (28). In a meta-analysis by Fernando et al., more than half of weight loss was related to a decrease in the fat mass (27). A systematic review by Chatson et al. on weight loss of more than 10 kg through diet and exercise showed that the loss of FFM accounted for 4.3–38.3% of the weight loss in an obesity intervention (29). Comparison of our results with previous research suggests that the effect of Ramadan fasting on the loss of FFM is at an acceptable and safe level.

Only few studies have directly investigated the impact of Ramadan fasting on the content of body water. A study by Alinezhad-Namaghi et al. showed that TBW had a significant, though small, reduction (0.75%) during Ramadan fasting (18). In the study by Leiper et al., by using a radiotracer technique, it was found that TBW was conserved in Ramadan despite a mild decrease in the daily water turnover (13). These findings may represent a new steady-state balance between fluid intake and water loss, leading to the relative preservation of TBW.

Considering the important role of phase angle in nutritional assessment, few studies have reported the impact of Ramadan fasting on phase angle. Similar to our study, Alinezhad-Namaghi et al., documented a significant reduction of about 1.7% in phase angle during Ramadan fasting (18). Further high-throughput studies are needed to evaluate the clinical importance and safe levels of alterations in the phase angle after Ramadan fasting.

### Possible explanations of the findings

Similar to intermittent fasting in Ramadan, short-term fasting of obese individuals was also associated with a loss of ICW content (18, 30). Several mechanistic explanations were proposed for the reduced ICW following fasting. It is estimated that about 2.7–4 g of water is bound to each 1 g of glycogen (31). Therefore, the reduction of the glycogen content of skeletal muscle cells after fasting can result in the extracellular shift of water and reduction of ICW. In contrast to Ramadan fasting, a study by Shiose et al. revealed that the ICW content increased in the lower limbs via carbohydrate loading and increased muscle glycogen stores (32). Aside from glycogen depletion, proteolysis and decreased activity of Na-K-ATPase ionic pump due to insulin deficiency may also alter the hydroelectrolytic balance of the intracellular compartment, resulting in a shift of water to the extracellular space (33). Confirming the redistribution of water after Ramadan fasting, we found a strong relationship between the reduction of 50-kHz phase angle and the increased ECW/TBW ratio. This correlation indicated an impairment of cell membrane integrity and the subsequent shift of water from the intracellular to the extracellular compartment.

### Study limitations

The present study had some limitations. First, a standardized method of bioimpedance analysis was applied in this study for the evaluation of body composition; however, we did not consider other variables related to the hydration status, such as urine output and osmolality, plasma osmolality, or serum creatinine. Second, data pertaining to physical activity, calorie intake, and dietary habits were not available to us; such data might explain the heterogeneity of our findings and account for the various metabolic effects of Ramadan fasting. Finally, we did not follow our participants after Ramadan; therefore, we cannot draw any definite conclusions about the reversibility of alterations induced by intermittent fasting during Ramadan.

## Conclusion

In this study, we found a significant, albeit small, amount of weight loss in the study samples after 20 consecutive days of fasting in Ramadan, which depended on the pre-fasting BMI. The loss of fat mass was the main contributor to the weight loss, and the amount of FFM loss was negligible. We also observed a significant decrease in the ICW content and an increase in the ECW/TBW ratio. These alterations in the body water compartments were accompanied by a decrease in the 50-kHz phase angle, which might indicate a shift of water from the intracellular to the extracellular compartment due to impaired cell membrane integrity. Future studies are needed to clarify the clinical correlation and safety of such alterations in body water compartments and composition following Ramadan fasting.

## Data availability statement

The raw data supporting the conclusions of this article will be made available by the authors, without undue reservation.

## Ethics statement

The studies involving humans were approved by Ethics Committee of Tehran University of Medical Sciences (IR.TUMS.IKHC.REC.1399.324). The studies were conducted in accordance with the local legislation and institutional requirements. The participants provided their written informed consent to participate in this study.

## Author contributions

MK, MA, and HM conceived and designed the study. MN, AS, AG, and MS collected the data. MS and AS analyzed the data. MS, MN, and HM wrote the first draft of manuscript. MK and MA revised the manuscript. All authors contributed, read, and approved the submitted version.

## Conflict of interest

The authors declare that the research was conducted in the absence of any commercial or financial relationships that could be construed as a potential conflict of interest.

## Publisher’s note

All claims expressed in this article are solely those of the authors and do not necessarily represent those of their affiliated organizations, or those of the publisher, the editors and the reviewers. Any product that may be evaluated in this article, or claim that may be made by its manufacturer, is not guaranteed or endorsed by the publisher.
